# Spousal Discrepancy in Perceived Importance of Care Needs Among Couples Managing Early-Stage Alzheimer’s Disease

**DOI:** 10.1093/geroni/igaf122.2075

**Published:** 2025-12-31

**Authors:** Eunju Lee, Kyungmin Kim, Meng Huo

**Affiliations:** Seoul National University, Seoul, Republic of Korea; Seoul National University, Seoul, Republic of Korea; University of California, Davis, Davis, California, United States

## Abstract

Understanding the care needs of people with Alzheimer’s disease (AD) is important for caregivers to make informed care decisions and prevent unmet needs of people living with AD (PLWD). Existing interventions have targeted promoting dementia knowledge in caregivers to help them better acknowledge PLWD’s common needs. Yet, caregivers’ empathy, their ability to understand and share PLWD’s emotions may be central as they assess their PLWD’s unique needs. This study examined discrepancies between PLWD and their spousal caregivers in perceived importance of PLWD’s care needs and how these discrepancies were associated with caregivers’ dementia knowledge and empathy (i.e., empathic concern, perspective taking, and personal distress). We also examined whether couple relationship quality moderated these associations. Data was from 65 couples coping with early-stage AD. PLWD self-reported the importance of physical, psychological, and social needs, and caregivers provided proxy reports on the perceived importance for PLWD. PLWD perceived addressing physical needs as more important than their caregivers, while the two parties showed agreements about perceived importance of PLWD’s psychological and social needs. Couples where caregivers reported greater empathic concern showed smaller discrepancies in importance of physical needs. Caregivers’ personal distress was negatively associated with the discrepancy in social needs. Caregivers’ dementia knowledge was not significantly associated with the discrepancy in importance across three types of needs, but dementia knowledge was positively associated with the discrepancy in perceived importance of psychological needs when relationship quality was low. The findings highlight the importance of caregivers’ emotional responsiveness and spousal dynamics in meeting PLWD’s needs.

